# Novel globular C1q domain-containing protein (PmC1qDC-1) participates in shell formation and responses to pathogen-associated molecular patterns stimulation in *Pinctada fucata martensii*

**DOI:** 10.1038/s41598-020-80295-0

**Published:** 2021-01-13

**Authors:** Xinwei Xiong, Chuyi Li, Zhe Zheng, Xiaodong Du

**Affiliations:** 1 Fishery College, Guangdong Ocean University, Zhanjiang, 524088 China; 2 Pearl Breeding and Processing Engineering Technology Research Centre of Guangdong Province, Zhanjiang, 524088 China; 3 Guangdong Science and Innovation Center for Pearl Culture, Zhanjiang, 524088 China; 4 Guangdong Provincial Engineering Laboratory for Mariculture Organism Breeding, Zhanjiang, 524088 China

**Keywords:** Immunology, Molecular biology, Genetics

## Abstract

The C1q protein, which contains the globular C1q (gC1q) domain, is involved in the innate immune response, and is found abundantly in the shell, and it participates in the shell formation. In this study, a novel gC1q domain-containing gene was identified from *Pinctada fucata martensii (P. f. martensii)* and designated as *PmC1qDC-1*. The full-length sequence of *PmC1qDC-1* was 902 bp with a 534 bp open reading frame (ORF), encoding a polypeptide of 177 amino acids. Quantitative real-time PCR (qRT-PCR) result showed that *PmC1qDC-1* was widely expressed in all tested tissues, including shell formation-associated tissue and immune-related tissue. *PmC1qDC-1* expression was significantly high in the blastula and gastrula and especially among the juvenile stage, which is the most important stage of dissoconch shell formation. *PmC1qDC-1* expression was located in the outer epithelial cells of mantle pallial and mantle edge and irregular crystal tablets were observed in the nacre upon knockdown of *PmC1qDC-1* expression at mantle pallial. Moreover, the recombined protein PmC1qDC-1 increased the rate of calcium carbonate precipitation. Besides, *PmC1qDC-1* expression was significantly up-regulated in the mantle pallial at 6 h and was significantly up-regulated in the mantle edge at 12 h and 24 h after shell notching. The expression level of *PmC1qDC-1* in mantle edge was significantly up-regulated at 48 h after LPS stimulation and was significantly up-regulated at 12 h, 24 h and 48 h after poly I:C stimulation. Moreover, *PmC1qDC-1* expression was significantly up-regulated in hemocytes at 6 h after lipopolysaccharide (LPS) and poly I:C challenge. These findings suggest that *PmC1qDC-1* plays a crucial role both in the shell formation and the innate immune response in pearl oysters, providing new clues for understanding the shell formation and defense mechanism in mollusk.

## Introduction

C1q protein, a versatile recognition protein, binds to a wide variety of immune and non-immune ligands^1,2^ via the C-terminal gC1q domain. Originally, C1q is involved in the classical complement pathway as the ligand-binding unit of the C1 complex^3,4^. As a versatile recognition protein, C1q recognizes many other ligands such as bacteria, viruses, parasites, mycoplasma, apoptotic cells, and pathogen-associated molecular patterns^5–8^. gC1q contains the globular subunit A, globular subunit B and globular subunit C^1,9^. Each subunit consisting of two five-stranded β-sheets is made of anti-parallel strands^10^. The Ca^2+^ ion binding site is present at the top of the C1q globular domain^10^ affecting the binding of C1q to diverse ligand^11^.

C1q domain-containing (C1qDC) proteins can be synthesized and secreted locally by various cell types, including macrophages, dendritic cells, fibroblasts, and mast cells that are ubiquitously distributed throughout the body^12^, to participate in the immune process. They can also be synthesized from specific tissues and organs such as microglial cells, glomerular and tubular cells, osteoclasts, and trophoblasts^13^ and are involved in the regulation of multiple cellular functions such as cell growth, clearance of the apoptotic cells, and promotion of cell adhesion^14^. When C1q is immobilized on a substrate or within a soluble immune-complex, it will enhance phagocytic function^15^. In humans, C1q deficiency causes systemic lupus erythematous^16^. During embryonic development, when the extravillous trophoblasts start to invade the decidua, C1q binds to the extracellular matrix and by interacting with cell surface-expressed receptors for the globular head of C1q and the associated α4β1 integrin, delivers an activation signal and promotes trophoblast migration^17^. C1q can regulate osteoclast development^18^. C1qDC proteins of bivalve mollusks can also recognize versatile ligands. *MgC1q* gene is involved in host defense in *Mytilus galloprovincialis*^19^. HcC1qDC6 is involved in innate immunity by directly binding to peptidoglycan (PGN) and LPS in *Hyriopsis cumingii*^20^. In *Crassostrea gigas*, CgC1qDC-6 mediates hemocyte phagocytosis and migration and can bind to various pathogen-associated molecular patterns (PAMPs), including LPS, PGN, mannose and poly I:C, and microorganisms^21^.

Related to the evolution of shells and the bivalve mollusk lifestyle^22,23^, the C1qDC gene of bivalve mollusks has undergone a massive expansion. The number of C1qDC genes in humans^24^, mice^25^, zebrafish^26^, and amphioxus^27^ ranges from 29 to 52. Strikingly, in mollusks, the number of C1qDC genes ranges from 168 to 476^22,28–30^. The large expansion of C1qDC causes its functions to have diversified. Many C1qDC genes of *P. f. martensii* are highly expressed in the mantle and pearl sac^31^, which is the shell formation associated tissue^32^*.* Wang discovered that PFMG4, which is highly homologous to C1q, could enhance osteoblast differentiation and serves as evidence of its participation in biomineralization^33^. Beside, shell matrix proteins (SMPs) containing C1q domain have been identified from many mollusks^34,35^. Although the C1q domain is an immune-related functional domain, growing body of evidence shows that in addition to immune function, C1qDC protein also contributes to shell formation. In most cases, the function of C1qDC only focuses on immunity or biomineralization. In *Hyriopsis cumingii*, a kunitz proteinase inhibitor participated in antimicrobial process duiring pearl sac formation and induced the overgrowth of calcium carbonate^36^. This show that there is no adversative relation in the roles between immune and biomineralization.Views on biochemical defense^34^, gastropod shell has been co-opted as a defense system against parasitic nematodes^37^. However, C1qDC protein, which has both immune function and biomineralization function, has not been reported yet. Therefore, it is necessary to further study the functions of C1qDC in the formation and defense of mollusk shell, which will help to understand the relationship between them.

In the present study, we aimed to examine the effect of PmC1qDC-1 on calcium carbonate precipitation rate and the expression pattern of *PmC1qDC-1* in different tissues, various larvae development stages, and shell damage repair stages to expose its biomineralized function and temporal response to immune challenge. Investigating the biomineralized and immunological function of *PmC1qDC-1* in *P. f. martensii* may provide new insights into the roles of C1qDC proteins in shell formation and defense mechanism of mollusks.

## Results

### Identification and sequence analysis of *PmC1qDC-1* gene

The full-length cDNA of *PmC1qDC-1* gene was 902 bp, containing an ORF of 534 bp, a 5′-untranslated region (UTR) of 288 bp, and a 3′-UTR of 50 bp with a 30 bp poly (A) tail (Supplementary Fig. 1). The cDNA sequence of *PmC1qDC-1* gene was deposited in GenBank (accession no. MT235265). *PmC1qDC-1* gene encodes 177 amino acids with a theoretical isoelectric point of 9.11 and a predicted molecular weight of 19.6 kDa. A signal peptide of 17 amino acid residues was predicted in the N-terminus of *PmC1qDC-1* by using SignalP-5.0 server. A globular C1q domain was located in the C-terminus according to the result of SMART analysis.

### Multiple sequence alignment and phylogenetic tree

**Figure 1:**
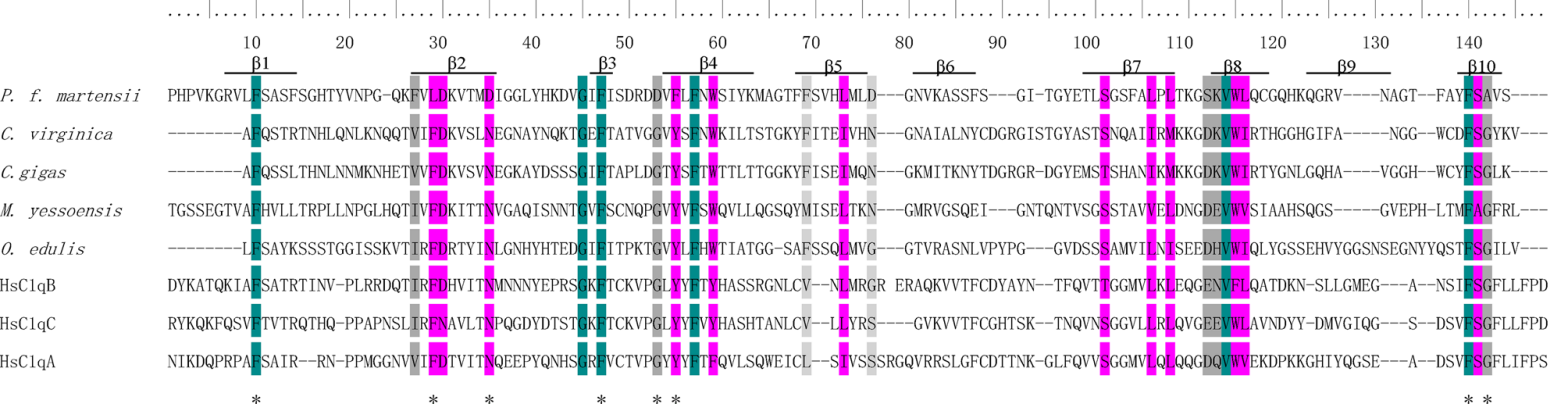
Multiple-sequence alignment of C1q-domain. *The eight conserved residues in the human gC1q domain; the blue background indicates conserved aa; the pink background indicates aa with strong similarity; the gray background indicates aa with weak similarity; the top numbers show the position of sequence alignment aa. The accession numbers of the sequences used in this alignment are as follows: *C. virginica* (XP_022296734.1), *C. gigas* (XP_011449189.1), *O. edulis* (AFK73703.1), and *M. yessoensis* (OWF54524.1). HsC1qB (NP_000482.3), HsC1qA (NP_057075.1), HsC1qC (NP_758957.2).

**Figure 2:**
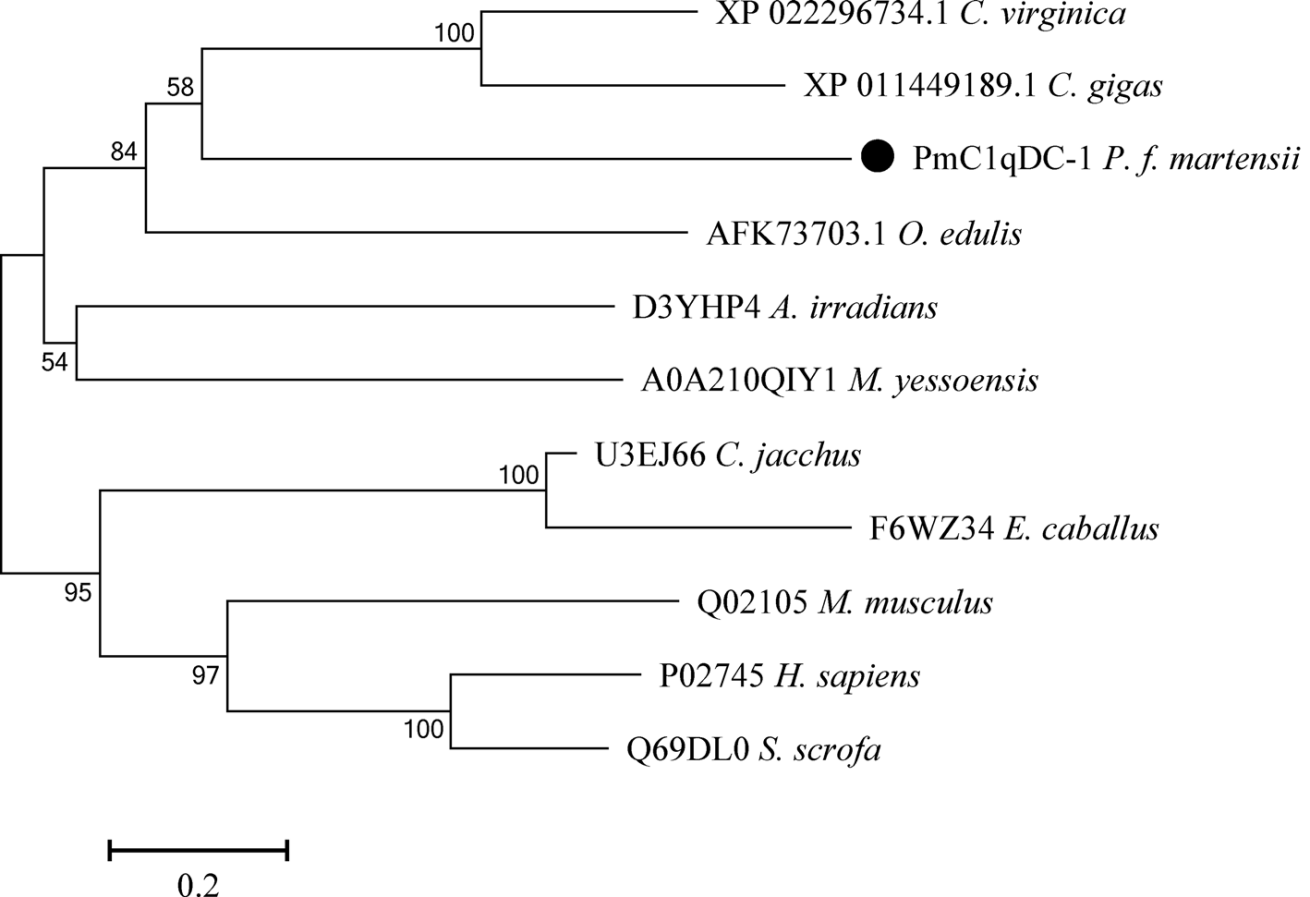
Neighbor-joining (NJ) tree of PmC1qDC-1 (●) with other C1qDC proteins. The tree was constructed by the NJ algorithm via MEGA 7 based on the multiple sequence alignment by muscles. Bootstrap values of 1000 replicates (%) are indicated for the branches. The scale bar corresponds to 0.2 estimated amino acid substitutions per site.

The deduced amino acid sequence of PmC1qDC-1 was homologous to the C1q family. NCBI BLAST analysis revealed that the deduced amino acid sequence of PmC1qDC-1 shared similarity with C1qDCs from other organisms, such as from *Crassostrea virginica* (28.57% identity with C1qDC), from *C. gigas* (26.20% identity with C1qDC), from *Ostrea edulis* (32.20% identity with C1qDC), and from *Mizuhopecten yessoensis* (29.7% identity with C1qDC)*.* The gC1q region of these C1qDCs was aligned. The 10-stranded β-sandwich in the gC1q domain of PmC1qDC-1 and the eight conserved residues in the human gC1q domain had been labeled (Fig. 1). The result of multiple alignments indicates that some of the eight amino acid residues conserved in the human C1q domain have been mutated in PmC1qDC-1. Phylogenetic tree was constructed to analyze the relationship between PmC1qDC-1 and C1qDC in other species (Fig. 2). The result indicates that PmC1qDC-1 clustered together with other C1qDC proteins from invertebrates, while the C1qDC in vertebrates were clustered together.

### Expression pattern of *PmC1qDC-1* in different tissues and in larvae development

**Figure 3:**
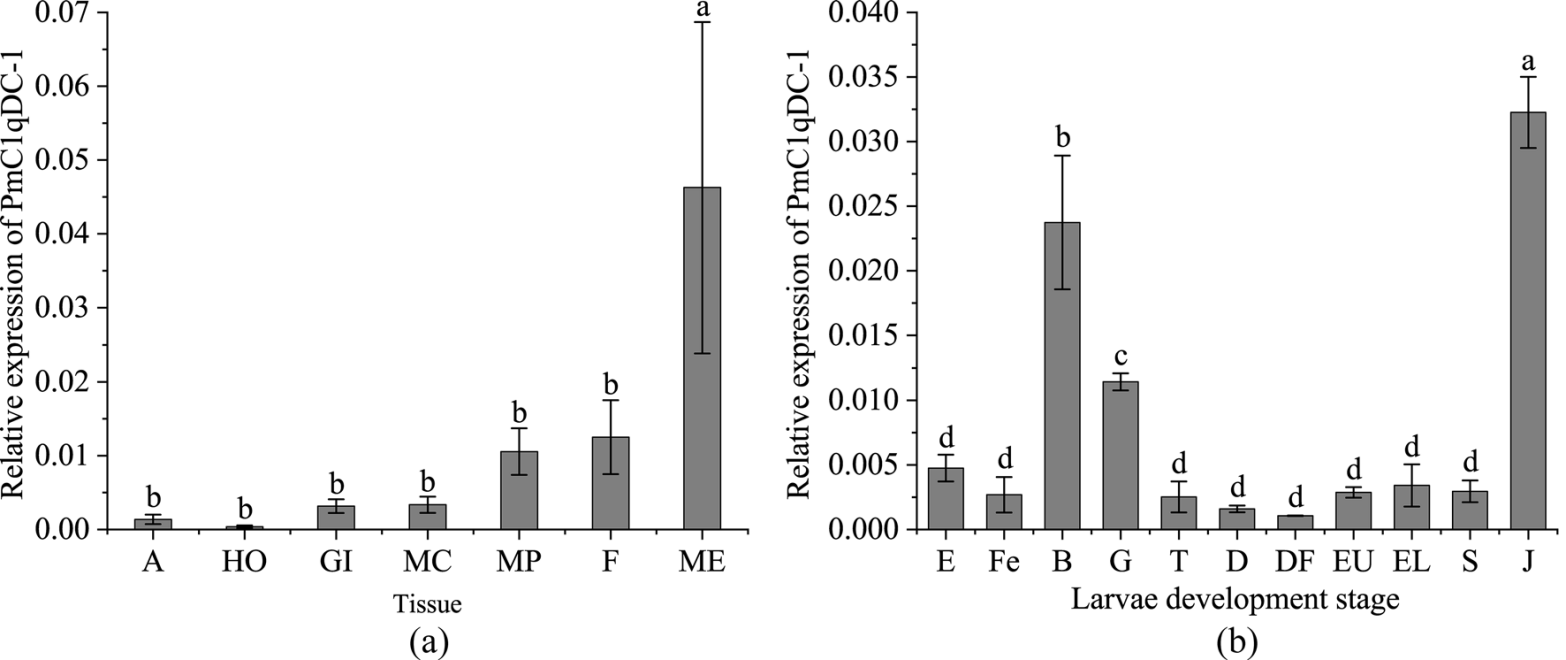
Expression pattern of *PmC1qDC-1* in different tissues and larvae development. (**a**) Expression pattern of *PmC1qDC-1* in different tissues. *A* adductor muscle, *HO* hemocytes, *GI* gill, *MC* mantle central, *MP* mantle pallial, *F* foot, *ME* mantle edge. (**b**) Expression pattern of *PmC1qDC-1* in larvae development. *E* egg, *Fe* fertilization, *B* blastula, *G* glastula, *T* trochophore stage, *D* D-stage larvae, *DF* D-stage larvae before feeding, *EU* early umbo larvae, *EL* eyed larvae, *S* spat, *J* juveniles. The averages of the groups with the different lower-case letters (a, b, c, d) are significantly different (P < 0.05). The averages of the groups with the same lower-case letters (a, b, c, d) are not significantly different (P > 0.05).

We detected the expression level of *PmC1qDC-1* in the mantle central, mantle pallial, mantle edge, foot, hemocytes, adductor muscle, and gill. *PmC1qDC-1* was constitutively expressed in all examined tissues, with the highest expression in the mantle edge (P < 0.05) and the lowest expression in hemocytes (Fig. 3a). We also detected the expression level of *PmC1qDC-1* at various larvae development stages (Fig. 3b). The result showed that *PmC1qDC-1* expression was significantly high in the blastula, and gastrula and among juveniles (P < 0.05).

### The spatial distribution of *PmC1qDC-1* in mantle

**Figure 4:**
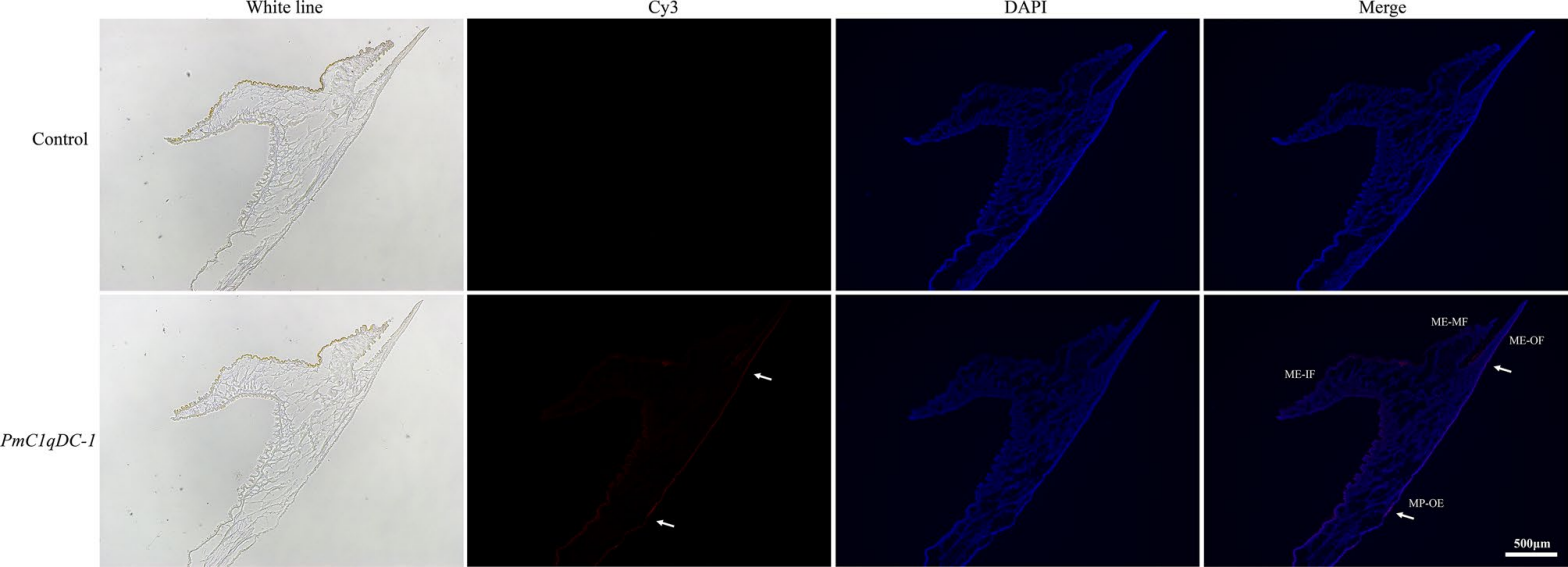
Fluorescent in situ hybridiazation results. *MP-OE* outer epithelium of mantle pallial, *ME-OF* outer fold of mantle edge, *ME-MF* middle fold of mantle edge, *ME-IF* inner fold of mantle edge. The blue fluorescence indicates nuclear dye signal. The red fluorescence and arrows indicate the positive signal of *PmC1qDC-1*. The white line in the image indicates the scale.

Fluorescent in situ hybridization (FISH) was performed to study the spatial distribution of *PmC1qDC-1* in mantle (Fig. 4). No obvious signal was found in control group. Strong hybridization signals were detected at the outer epithelial cells of mantle pallial and mantle edge while a faint signal was detected at the inner epithelial cells of mantle pallial.

### Expressed PmC1qDC-1 fusion protein

**Figure 5:**
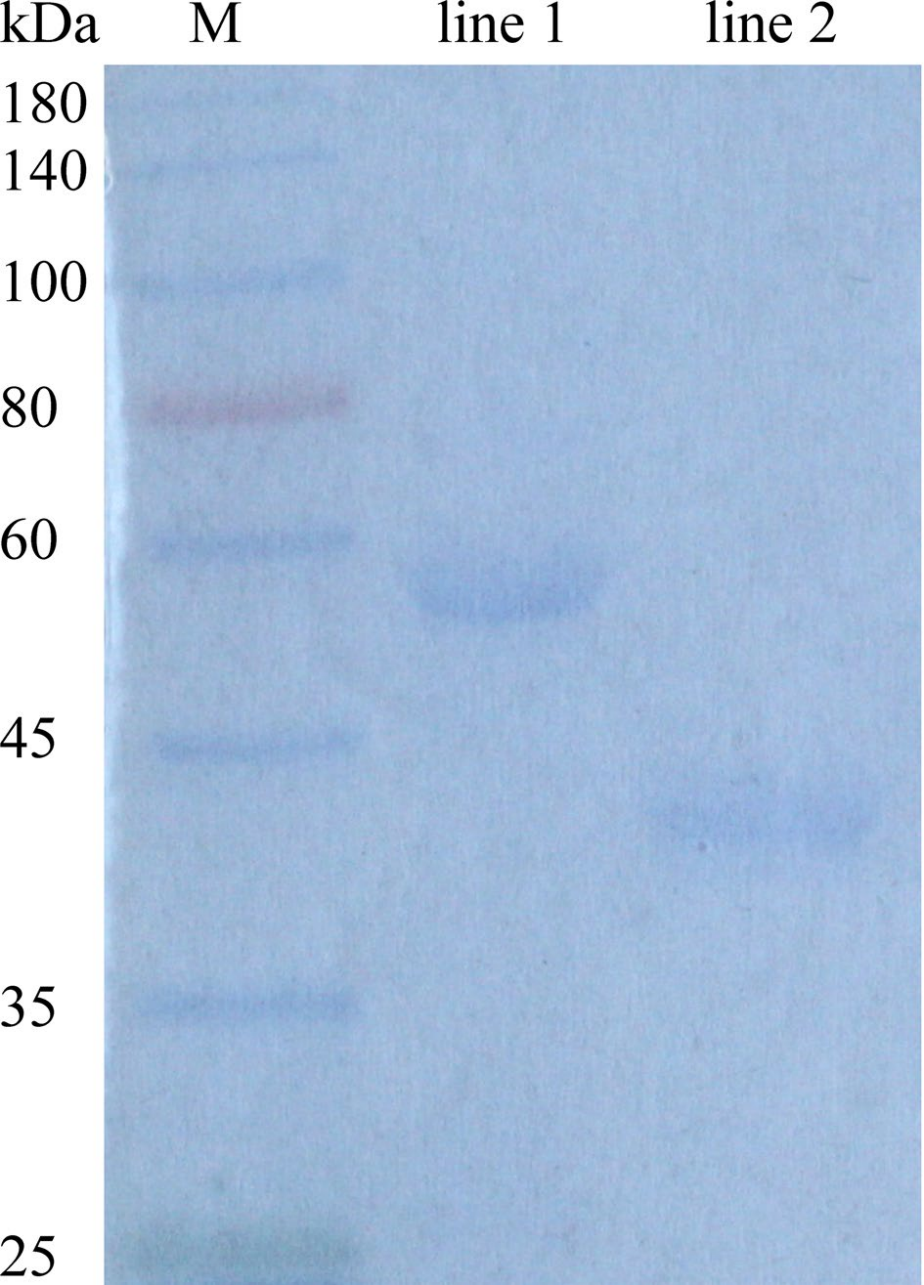
SDS-PAGE gel electrophoresis of PmC1qDC-1 and MBP protein. *M* molecular mass markers, *lane 1* purified PmC1qDC-1 protein, *line 2* MBP protein; the molecular mass (kDa) is shown on the left of the gel.

Recombinant PmC1qDC-1 with maltose-binding protein (MBP) tag was expressed under 1 mM isopropyl 1-thio-β-d-galactopyranoside (IPTG) induction. The main band in the figure of SDS-PAGE gel (Fig. 5) was consistent with the calculated value (PmC1qDC-1 16 kDa plus MBP 40 kDa).

### PmC1qDC-1 increased calcium carbonate precipitation and contributed to shell formation

**Figure 6:**
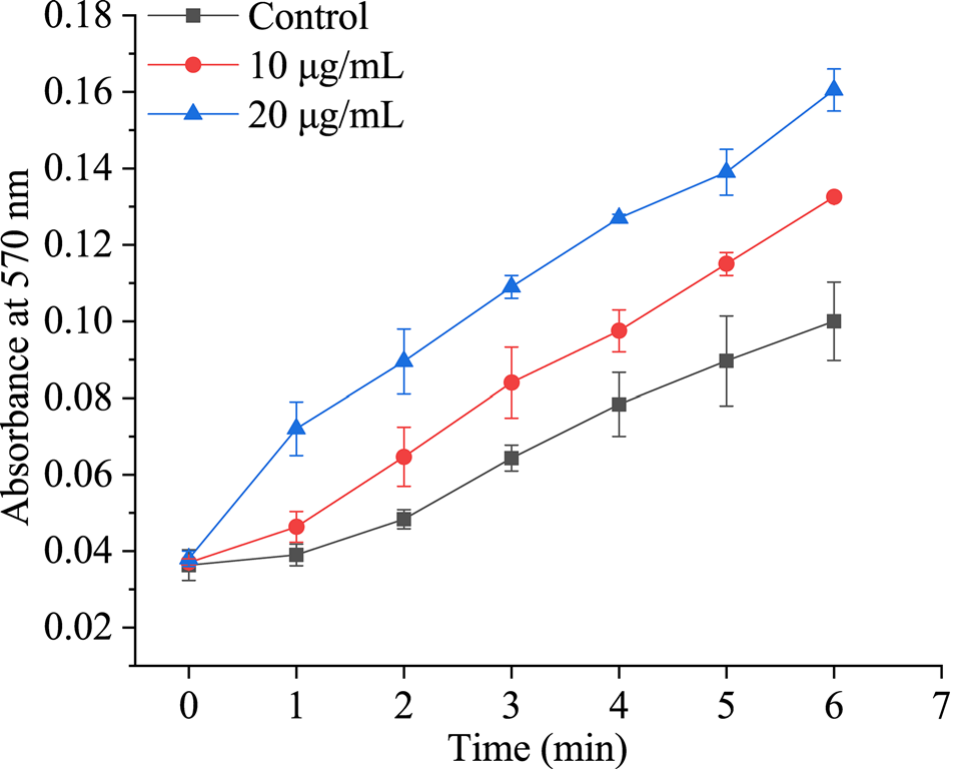
PmC1qDC-1 increases calcium carbonate precipitation. ▲, 20 μg/mL PmC1qDC-1; ●, 10 μg/mL PmC1qDC-1; ■, 20 μg/mL MBP as control.

In vitro, the effect of PmC1qDC-1 on the precipitation rate was determined via a calcium carbonate precipitation experiment. Compared with the control, PmC1qDC-1 increased the precipitation rate, and this effect was concentration dependent (Fig. 6).

**Figure 7:**
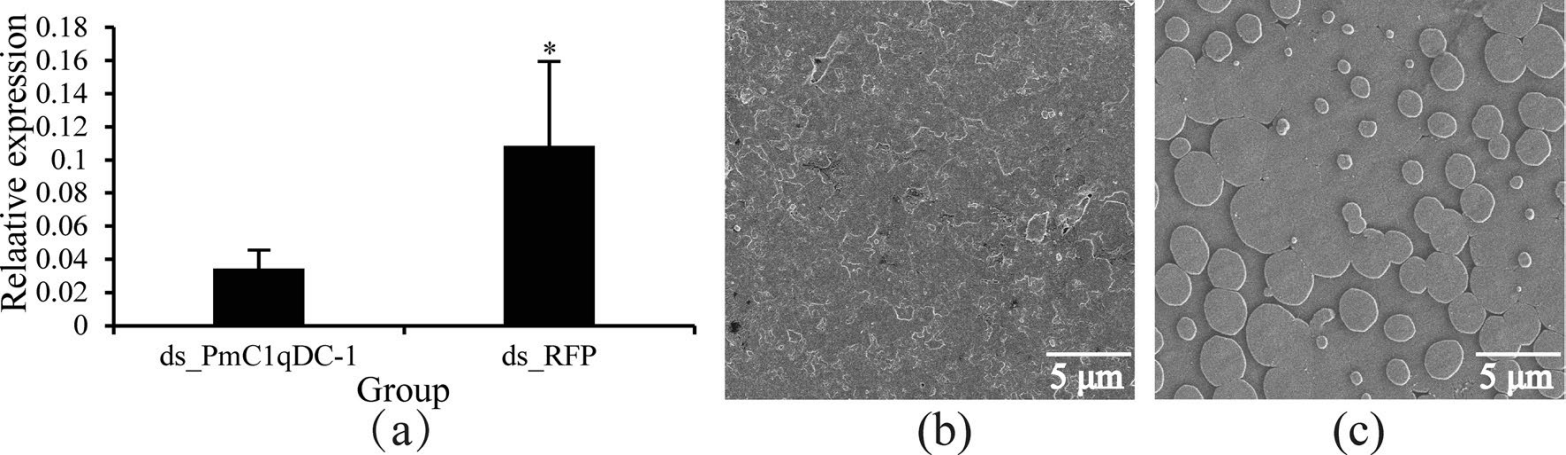
Effect of *PmC1qDC-1* on shell formation by RNA interference (RNAi). (**a**) Relative expression of *PmC1qDC-1* at the mantle pallial after RNAi. (**b**) Inner surface microstructure of the nacre layer in the experimental group. (**c**) Inner surface microstructure of the nacre layer in control group. *Significant difference. The white line in the image indicates the scale.

In vivo, we knocked down *PmC1qDC-1* expression by injecting ds_PmC1qDC-1. The mRNA expression levels of *PmC1qDC-1* in the mantle pallial significantly decreased after treatment (Fig. 7a). We observed the inner surface microstructure of the nacre layer under a scanning electron microscope. Disordered crystal growth was observed in the experimental group (Fig. 7b), while the crystal growth on the control group was normal (Fig. 7c).

### Expression pattern of *PmC1qDC-1* after shell notching

**Figure 8:**
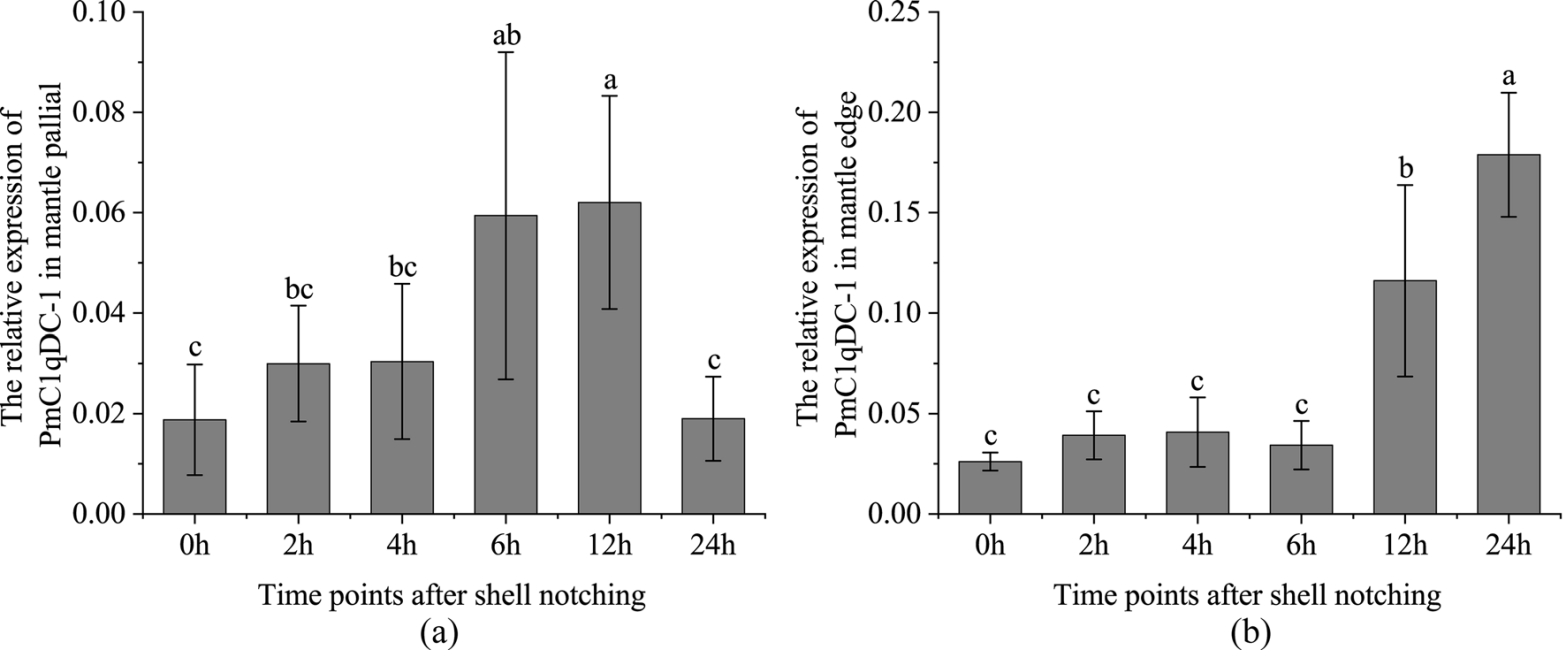
Expression pattern of *PmC1qDC-1* in shell damage repair process. (**a**) Expression pattern of *PmC1qDC-1* at the mantle pallial after shell notching. (**b**) Expression pattern of *PmC1qDC-1* at the mantle edge after shell damage. The averages of the groups with the different lower-case letters (a, b, c) were significantly different (P < 0.05). The averages of the groups with the same lowercase letters (a, b, c) are not significantly different (P > 0.05).

We investigated the expression patterns of *PmC1qDC-1* in shell damage repair. *PmC1qDC-1* expression in the mantle pallial was gradually up-regulated after shell injury and significantly up-regulated at 6 h and 12 h compared with that at 0 h (Fig. 8a). *PmC1qDC-1* gene was significantly overexpressed at the mantle edge 12 h and 24 h after shell notching (Fig. 8b).

### *PmC1qDC-1* response to PAMPs stimulation

**Figure 9:**
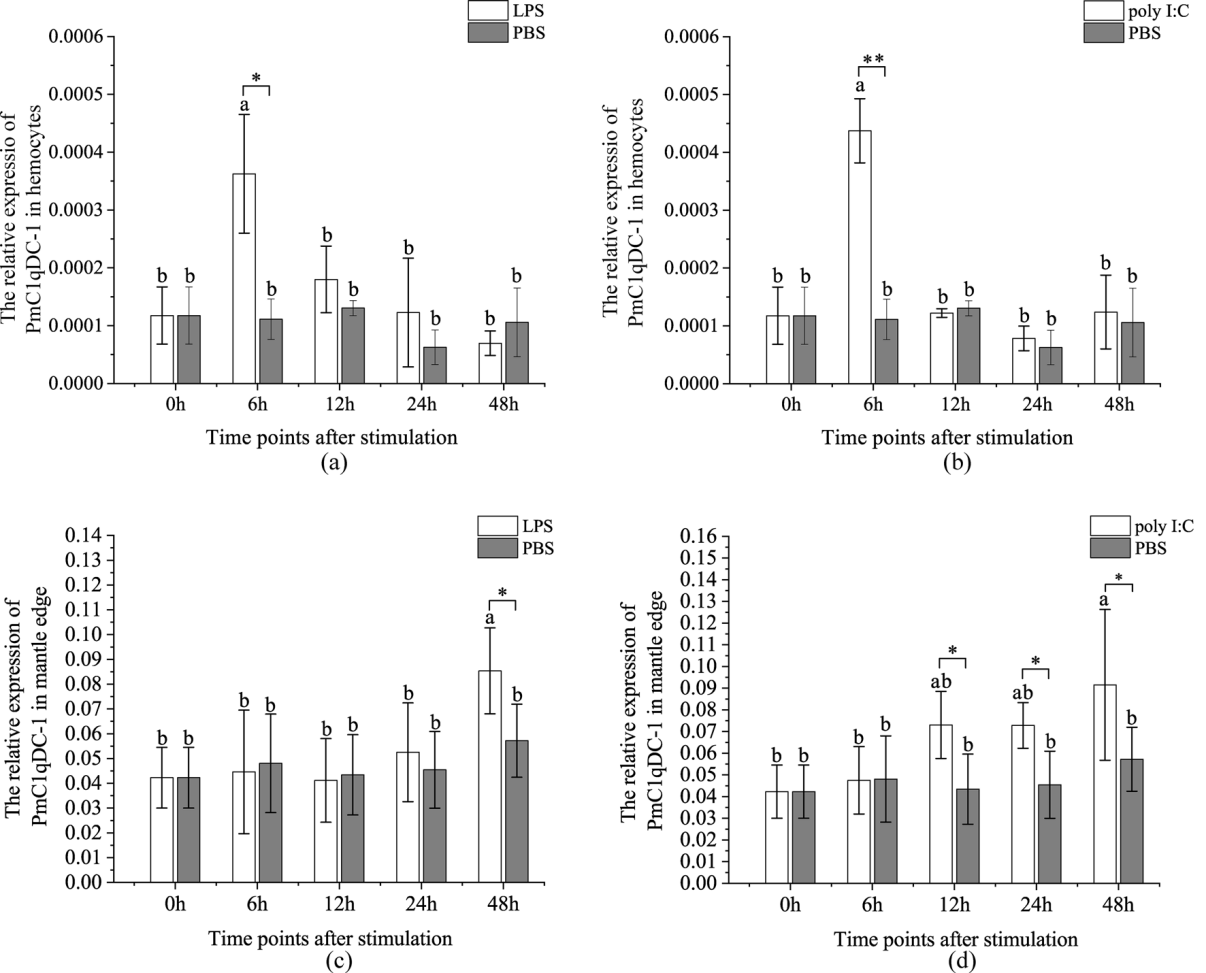
Expression pattern of *PmC1qDC-1* after immune challenge. (**a**) Expression pattern of *PmC1qDC-1* in hemocytes after LPS injection. (**b**) Expression pattern of *PmC1qDC-1* in hemocytes after poly I:C injection. (**c**) Expression pattern of *PmC1qDC-1* in mantle after LPS injection. (**d**) Expression pattern of *PmC1qDC-1* in mantle poly I:C injection. The averages of the groups with the different lower-case letters (a, b, c) are significantly different (P < 0.05). The averages of the groups with the same lower-case letters (a, b, c) were not significantly difference (P > 0.05). *Significant difference in expression between the two groups at the same time point (P < 0.05). **That the expression levels between the two groups are extremely significant at the same time point (P < 0.01).

The response of the *PmC1qDC-1* gene to LPS and poly I:C stimulation was detected by quantitative real-time PCR (qRT-PCR) (Fig. 9). β-actin was used as the referent gene to determine relative expression levels. The expression level of *PmC1qDC-1* in hemocytes was significantly up-regulated at 6 h after injecting LPS (Fig. 9a). After challenge with poly I:C for 6 h, *PmC1qDC-1* expression in hemocytes was significantly up-regulated (Fig. 9b). We also detected the expression pattern of *PmC1qDC-1* in mantle edge after LPS and poly I:C stimulation. The expression level of *PmC1qDC-1* in mantle edge was significantly up-regulated at 48 h after LPS stimulation (Fig. 9c). After challenge with poly I:C for 12 h, 24 h, and 48 h, the expression level of *PmC1qDC-1* in mantle edge was significantly up-regulated compared with control group (Fig. 9d).

## Discussion

C1q is the key component of complement system containing the typical C-terminal globular C1q domain. C1qDC protein can recognize many ligands via the gC1q domain with the common binding sites^21,38^. In the present study, we identified a novel gC1q gene from *P. f. martensii* and designated it as *PmC1qDC-1*. PmC1qDC-1 contains a typical C-terminal globular C1q domain with 10 β-stand sheets and is considered as a member of the C1qDC protein family. All invertebrate C1qDCs, including those from *C. gigas*, *M. yessoensis*, *C. virginica*, *O. edulis*, and *A. irradians*, were grouped into a single branch. Thus, *PmC1qDC-1* and other invertebrate C1qDCs may have evolved from the same ancestral gene, and subsequent selection pressure then led to the diversification of those genes within the invertebrate lineage. Multiple sequence alignment also supports this hypothesis. PmC1qDC-1 has low sequence similarity to C1qDC in other mollusks (26.2–32.2%), and some of the eight conserved amino acids^39^ in the human C1q domain had been mutated in PmC1qDC-1; thus these amino acids may have mutated to accommodate versatile attributes of function.

We detected the expression pattern of *PmC1qDC-1* in seven tissues. Constitutive expression in all of these tissues suggested the possibility of *PmC1qDC-1* performing various functions in different tissues. Although the C1q domain is an immune-related domain, it also functions as SMPs existing in shell^35^. The high expression of *PmC1qDC-1* in the mantle suggests that it may be involved in shell formation. The highest expression of *PmC1qDC-1* in juveniles indicats that *PmC1qDC-1* participats in the formation of dissoconch shell, which is the normal adult shell structure^40^. The mantle is the most important tissue involved in biomineralization and mantle pallial is responsible for the formation of nacreous layer. The strong hybridization signals located in the outer epithelial cells of mantle pallial indicates that *PmC1qDC-1* participated in nacreous formation. Moreover, in vitro, PmC1qDC-1 can increase the calcium carbonate precipitation rate. Especially, disordered crystal growth was observed in the nacre upon knockdown of *PmC1qDC-1* expression at the mantle pallial. This result indicats that *PmC1qDC-1* is directly involved in nacre formation. The C1q globular domain consists of spherical heterotrimeric with Ca^2+^ ion bound at the top^2^. Therefore, *PmC1qDC-1* may participate in nacre formation by accumulating Ca^2+^ ions. However, the mechanism through which *PmC1qDC-1* participates in nacre formation requires further investigation.

gC1q protein is common in bivalves. For example, 167 of the 168 C1qDC gene models in *Mediterranean mussel* are gC1q proteins^28^. Numerous invertebrate gC1q proteins are involved in the immune process. For example, MgC1q acts as a pattern-recognition molecule that can recognize pathogens during innate immune responses in *M. galloprovincialis*^19^. AiC1qDC-1 of scallops can agglutinate fungi and has mannose and PGN binding sites at its gC1q domain^41^. *PmC1qDC-1* is extremely highly expressed at the mantle edge, indicating that it may be involved in resisting bacterial and pathogenic invasion. Besides the key roles of the mantle edge in shell formation, it also plays important roles in direct immune defense during exposure to seawater condition and even in shell damage and parasitic invasion in bivalves. For example, the mantle edge of *C. gigas* can secrete defensin in response to pathogen colonization^42^. The expression level of *PmC1qDC-1* in mantle edge was significantly up-regulated at 48 h after LPS stimulation. After challenge with poly I:C for 12 h, 24 h, and 48 h, *PmC1qDC-1* was significantly-high expressed in mantle edge compared with control group. The expression fluctuation of *PmC1qDC-1* after LPS and poly I:C stimulation indicates that it is involved in immune defense. *PmC1qDC-1* was significantly up-regulated in the mantle pallial at 6 h and 12 h post of shell notching while it was significantly up-regulated at mantle edge at 12 h and 24 h after shell damage. At the shell regeneration stage, shell damage will accelerate SMPs secretion^43^, and the organic membrane where the initiation of crystal deposition occurrs has formed near the nick at 6 h after shell notching^43,44^. Moreover, the mantle tissue will retract and increase the zone of tissue exposed to seawater due to the “V” nick, which will easily result in a second injury due to bacterial and pathogen infection^43^. Thus, *PmC1qDC-1* may participate in shell regeneration and in the immune process of shell damage.

C1qDC proteins, which are pattern recognition molecules, rely on their gC1q domain to recognize a variety of self and non-self ligands, including a vast range of PAMPs of bacteria, yeast, viruses, and parasites^20,45^. The shell notching experiment indicates that *PmC1qDC-1* participates in the immune process. The hemocytes, the most critical immune organ, play a central role in the recognition of exogenous agents and in the defense against bacterial invasion in mollusks^46^. However, the expression level in hemocytes was relatively low. We speculate that, similar to HcC1qDC6^20^, *PmC1qDC-1* expression in hemocytes may be induced under stress. As such, the challenge experiment of LPS and poly I:C was performed to further understand the immune-related functions of *PmC1qDC-1* in pearl oysters. *PmC1qDC-1* was significantly up-regulated in the hemocytes at 6 h after injecting LPS, and the same expression pattern was present in the poly I:C treatment group. This finding indicates that *PmC1qDC-1* is involved in the immune response against pathogen invasion.

In this work, we identified a novel gC1q gene named *PmC1qDC-1*. *PmC1qDC-1* participates in nacre formation by increasing the calcium carbonate precipitation. *PmC1qDC-1* may be involved in shell regeneration and immune response after shell injury. *PmC1qDC-1* gene can quickly respond to LPS and poly I:C challenge. These findings revealed that it participates in shell formation and the innate immune response in *P. f. martensii*.

## Materials and methods

### Experimental animals

Adult pearl oysters (2 years old) were obtained from Houhong Xuwen, Zhangjiang,Guangdong Province, China. The animals were cultured at 25–28 ℃ indoor breeding tanks with seawater for 2 days before the experiment.

The sample of larvae at different development stages was the same used in previous genomic research^32^.

### RNA isolation and cDNA synthesis

Various tissues containing the mantle central, mantle pallial, mantle edge, foot, hemocytes, adductor muscle, and gill were separated from the pearl oysters. Total RNA was isolated through TRIzol method following the protocol we submitted to protocols.io before (https://dx.doi.org/10.17504/protocols.io.9qgh5tw). Absorbance at OD260/OD280 was measured using a NanoDrop ND 1000 spectrophotometer (ThermoFisher Scientific Inc, Waltham, MA, USA) to confirm the RNA quantity. RNA integrity was determined by fractionation on 1.0% agarose gel. cDNA was synthesized using an M-MLV reverse transcriptase (Promega, USA). In brief, 500 ng of RNA and 1 μL of random primers were mixed, and RNase free water was added until 6 μL was reached, and the mix was incubated at 70 ℃ for 10 min. During this time, 2 μL of 5× M-MLV buffer, 0.5 μL of 10 mM dNTPmixture, 0.25 μL of RNase inhibited, and 0.5 μL of RTase M-MLV were mixed and added with RNase free water to reach 4 μL. Finally, the two reaction solutions mixed well and incubated at 42 ℃ for 1 h and 70 ℃ for 15 min. The first-strand cDNA was synthesized using SMARTer Rapid amplification of cDNA ends (RACE) 5′/3′ kit (TaKaRa, Dalian, China) in accordance with the manufacturer’s instructions.

### Gene cloning of *PmC1qDC-1* and sequence analysis

In the present study, the full-length cDNA of *PmC1qDC-1* was obtained using RACE. The procedure for PCR is listed as follows: 95 ℃ for 5 min, 38 cycles at 98 ℃ for 15 s, 60 ℃ for 30 s, 72 ℃ for 2 min, and 72 ℃ for 10 min. The PCR product was gel purified and cloned into a PMD 18-T simple vector (TaKaRa, Dalian, China). The base sequence was obtained via Sanger sequencing. All the primers used in the study were listed in the Table 1.

**Table 1:** Names and sequences of primers used in this study. Note: The sequences underlined are the sequence of the T7 promoter.

Primer name	Primer sequence (from 5′ to 3′)	Application
PmC1qDC-1-F	ATGGAGACAGGTTTACCTCTTATA	CDS clone
PmC1qDC-1-R	CTACAGTTTGTATCCAGAAAATGCT	CDS clone
NUP	GCAGTGGTATCAACGCAGAGT	RACE
UPM-short	CTAATACGACTCACTATAGGGC	RACE
UPM-long	CTAATACGACTCACTATAGGGCAAGCAGTGGTATCAACGCAGAGT	RACE
PmC1qDC-1-5′-outer	CACTGAAAAATGTCCCTGCCATCTTAT	RACE
PmC1qDC-1–5′-inner	CGTAAGTATGACCCGAAAAGGATG	RACE
PmC1qDC-1-3′-outer	TGATGTTGGACGGGAATGTGAAAG	RACE
PmC1qDC-1-3′-inner	ACATAAACAAGGAAGAGTAAATGCTGGGAC	RACE
PmC1qDC-1-qPCR-F	GAAAGTCTGTAAACCTGGCACC	qRT-PCR
PmC1qDC-1-qPCR-R	ACATTCCCGTCCAACATCAA	qRT-PCR
GAPDH-F	GCAGATGGTGCCGAGTATGT	qRT-PCR
GAPDH-R	CGTTGATTATCTTGGCGAGTG	qRT-PCR
β-Actin-F	CGGTACCACCATGTTCTCAG	qRT-PCR
β-Actin-R	GACCGGATTCATCGTATTCC	qRT-PCR
PmC1qDC-1-T7-F	ACTCACTAATACGACTCACTATAGGGATGGAGACAGGTTTACCTCTTATA	RNAi
PmC1qDC-1-T7-R	ACTCACTAATACGACTCACTATAGGGCTACAGTTTGTATCCAGAAAATGCT	RNAi
ISH576.501-F	CCTTTTCGGGTCATACTTACG	FISH
ISH576.501-R	ACTCACTAATACGACTCACTATAGGGCCAGCATTTACTCTTCCTTGTTTA	FISH
PmC1qDC-1-XhoI	TTTTCAGGGTCTCGGATCCCTCGAGGCTTTTTCCTTGAAATACGA	Subclone
PmC1qDC-1-EcoRI	GGTGGTGGTGGTGGGTCTCGAATTCAAGGCTCACAGCAGAGAAGT	Subclone

We used the ORF Finder tool (https://www.ncbi.nlm.nih.gov/orffinder/) and SignalP-5.0 Server (http://www.cbs.dtu.dk/services/SignalP/) to obtain the ORF region and signal peptide of *PmC1qDC-1*. Domain information of *PmC1qDC-1* was obtained through the Simple Modular Architecture Research Tool (http://smart.embl-heidelberg.de/smart/show_motifs.pl). Clustal Omega website tool (https://www.ebi.ac.uk/Tools/msa/clustalo/) was used to align the protein sequences. Evolutionary relationship of PmC1qDC-1 and other orthologs was build up by MEGA 7.

### qRT-PCR and statistical analysis

We used the qRT-PCR to test *PmC1qDC-1* expression levels. The mix reagent was from DyNAmo Flash SYBR Green qPCR kit (ThermoFisher Scientific). The qRT-PCR experiment of *PmC1qDC-1* was carried out on Applied Biosystems 7500/7500 Fast Real-Time System (Applied Biosystems, Foster City, CA, USA). The PCR program was as follows: 95 °C for 5 min, 40 cycles at 95 °C for 30 s, 60 °C for 15 s, 72 °C for 15 s. The relative expression levels of reference genes (β-actin and GAPDH) and *PmC1qDC-1* were calculated through the 2^−ΔCT^ method.

Significance was analyzed using SPSS 22.0 (IBM, Chicago, IL, USA). The expression levels of *PmC1qDC-1* at the tissues, different development stages, and different time points of shell notching were analyzed using one-way ANOVA. Differences in *PmC1qDC-1* expression between the two groups were evaluated using the T-test. The significant level for these analyses was set at P < 0.05.

### Fluorscent in situ hybridization experiment

RNA probes of *PmC1qDC-1* were synthesized in vitro by using T7 RNA polymerase and digoxigenin (DIG) RNA Labeling Mix. Integrity of *PmC1qDC-1* RNA probes was confirmed by using 1% agarose gel electrophoresis. Concentration and purity of *PmC1qDC-1* RNA probes were detected by using a nucleic acid quantifier. The mantle of *P. f. martensii* was separated and fixed 2 h in 4% paraformaldehyde containing 0.1% diethyl dicarbonate (Sangon Biotech). Then the mantle tissues were washed with phosphate buffer saline (PBS) for three times and embedded in the medium called optimal cutting temperature (O. C. T.). Finally, the embedded samples were made into slices with 10 μm thickness via the instrument of LEICA CM3050 S. Fluorescent in situ hybridization was constructed through the instructions of Enhanced Sensitive ISH Detection Kit IV (CY3) (BOSTER). The detailed protocol was referred to the file we submitted to protocols.io before (10.17504/protocols.io.9qhh5t6). The fluorescence signals were observed under microscope (Nikon ECLIPSE Ni, DS-Ri2).

### *PmC1qDC-1* function interference experiment

RNAi was used to knockdown the expression level of *PmC1qDC-1* following the protocol of Hao et al.^47^. In brief, double-stranded RNA (dsRNA) of *PmC1qDC-1* (ds_PmC1qDC-1) and Red Fluorescent Protein gene (ds_RFP) were synthesized and purified by using a T7 High-Efficiency Transcription kit (TransGen Biotech, JT101) and EasyPure RNA Purification kit (TransGen Biotech, ER701) correspondingly. 100 µL ds_PmC1qDC-1 were injected into the adductor muscle of *P. f. martensii*, while 100 µL ds_RFP were injected at the control group. Mantle pallial was collected, put in liquid nitrogen at the 4 days post second injection and then frozen samples were transferred in − 80 °C freezer. The shells of all groups were cut into a small piece (0.5 cm × 1.5 cm) containing the transition region of the nacre and prismatic layer. The shell samples were clean with ultrapure water and air dried. The inner surface of nacre layer near the transition was observed under a scanning electron microscope (JSM-6300 LV) to obtain the image of ultrastructure.

### Prepared recombinant protein of PmC1qDC-1

Recombinant PmC1qDC-1 protein was prepared in cooperation with Abmart. *PmC1qDC-1* gene sequence without signal peptide was subcloned to the vector modified from (pET30a) by Abmart, and the recombinant plasmid was transformed using Rosetta Competent Cell for expression. Transformed cells were cultured in Luria–Bertani medium (with 50 mg/mL kana^+^) at 37 ℃ 200 rpm, induced with 1 mM IPTG when OD600 reached 0.5–0.8, and cultured at the same condition for another 4 h. The cell pellet was collected after centrifugation at 6000*g* for 5 min at 4 ℃ and washed three times with PBS. The harvested cells were resuspended with PBS and subjected to ultrasonication. The protein was purified using Amylose Resin (NEB). The wash fractions with 10 mM maltose were collected, boiled and loaded onto an SDS-PAGE gel. The purified protein was desalted by dialysis and freeze dried for storage. The protein concentration was measured by using BCA assay kit (Sangon Biotech).

### Calcium carbonate precipitation assay

The effect of PmC1qDC-1 on the rate of calcium carbonate precipitation was tested following the method of Dong et al.^48^. The control was 20 μg/mL MBP. The 10 μL of sample solution and 100 μL of calcium chloride (100 Mm, pH 8.5) were added to 96-well plates and mixed completely. Then, 100 μL of 100 mM sodium bicarbonate was added to the mixed solution quickly. The formation of calcium carbonate precipitate was tested by recording the absorbance at 570 nm every 1 min by using a multimode plate reader (EnSpire, PerkinElmer).

### Shell notching and PAMP stimulation

The notching assays were performed on 35 normal pearl oysters. Thirty normal pearl oysters were selected and a “V” shaped notch was cut on the shell until the nacreous layer was reached. The mantle edge and mantle pallial of every five pearl oysters were collected at 2 h, 4 h, 6 h, 12 h, and 24 h after damage, and the mantle edge of five pearl oysters (no notching) was harvested at 0 h.

The pearl oysters were randomly divided evenly into three groups (36 individuals for each group), and 100 μL (10 μg/mL) each of LPS, poly I:C, and PBS were injected into the adductor muscle. The pearl oysters were cultured in an indoor container with sea water at approximately 25 ℃. Hemocytes and mantle edge were harvested at 6 h, 12 h, 24 h, and 48 h after stimulation. In blank group, hemocytes and mantle edge were collected from the pearl oyster without stimulation at 0 h.

## Supplementary Information

Supplementary Figure 1.
