# Supplementary material for: Novel globular C1q domain-containing protein (PmC1qDC-1) participates in shell formation and responses to pathogen-associated molecular patterns stimulation in Pinctada fucata martensii
Source: Sci Rep. 2021 Jan 13;11:1105. doi: 10.1038/s41598-020-80295-0 (PMC7806589; doi:10.1038/s41598-020-80295-0)
Supplement: Supplementary file 1 — Supplementary Figure 1. [file 41598_2020_80295_MOESM1_ESM.docx]

**Supplementary information**

**Novel globular C1q domain-containing protein (PmC1qDC-1) participates in shell formation and responses to** **pathogen-associated molecular patterns stimulation in *Pinctada fucata martensii***

**Xinwei Xiong^1^ ChuYi Li^1^ Zhe Zheng^1,2,3,4, *^ Xiaodong Du^1,2,3,4 *^**

1 Fishery College, Guangdong Ocean University, Zhanjiang 524088, China;

2 Pearl Breeding and Processing Engineering Technology Research Centre of Guangdong Province, Zhanjiang, 524088, China

3. Guangdong Science and Innovation Center for Pearl Culture, Zhanjiang, 524088, China

4. Guangdong Provincial Engineering Laboratory for Mariculture Organism Breeding, Zhanjiang, 524088, China

* Correspondence: [gdhddxd@hotmail.com](mailto:gdhddxd@hotmail.com) (Xiaodong Du) and haidazhengzhe@163.com (Zhe Zheng); Tel.: +86-759-238-3346; Fax: +86-759-238-2404


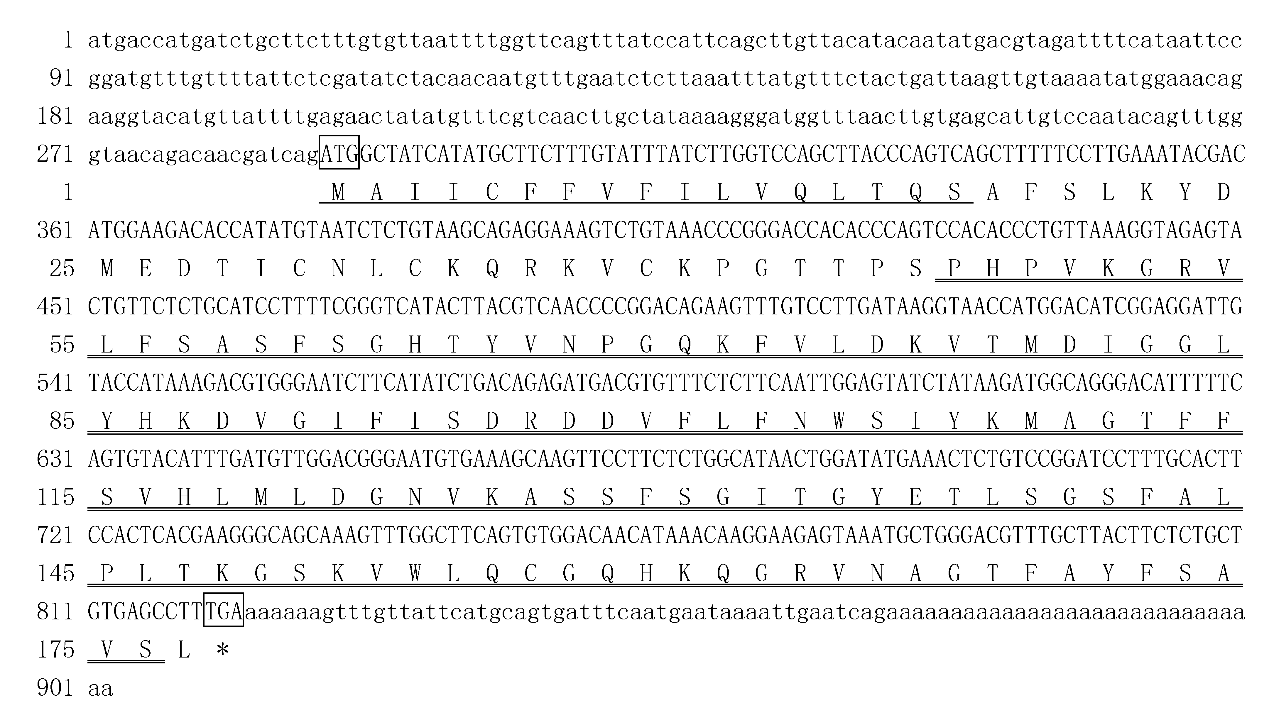


**Supplementary Figure 1** Nucleotide and amino acid sequence of *PmC1qDC-1*. The 5′-UTR and 3′-UTR are indicated with small letters. The 17 amino acid signal peptide and the C1q domain are indicated with an underline and a double underline, respectively. The nucleotide with a frame represents the start and stop codons.
